# Visualizing learner engagement, performance, and trajectories to evaluate and optimize online course design

**DOI:** 10.1371/journal.pone.0215964

**Published:** 2019-05-06

**Authors:** Michael Ginda, Michael C. Richey, Mark Cousino, Katy Börner

**Affiliations:** 1 Department of Intelligent Systems Engineering, School of Informatics, Computing, and Engineering, Indiana University, Bloomington, Indiana, United States of America; 2 The Boeing Company, Everett, Washington, United States of America; University of Westminster, UNITED KINGDOM

## Abstract

Learning analytics and visualizations make it possible to examine and communicate learners’ engagement, performance, and trajectories in online courses to evaluate and optimize course design for learners. This is particularly valuable for workforce training involving employees who need to acquire new knowledge in the most effective manner. This paper introduces a set of metrics and visualizations that aim to capture key dynamical aspects of learner engagement, performance, and course trajectories. The metrics are applied to identify prototypical behavior and learning pathways through and interactions with course content, activities, and assessments. The approach is exemplified and empirically validated using more than 30 million separate logged events that capture activities of 1,608 Boeing engineers taking the MITxPro Course, “Architecture of Complex Systems,” delivered in Fall 2016. Visualization results show course structure and patterns of learner interactions with course material, activities, and assessments. Tree visualizations are used to represent course hierarchical structures and explicit sequence of content modules. Learner trajectory networks represent pathways and interactions of individual learners through course modules, revealing patterns of learner engagement, content access strategies, and performance. Results provide evidence for instructors and course designers for evaluating the usage and effectiveness of course materials and intervention strategies.

## Introduction

In the information age, skills and knowledge required to perform professional jobs are changing rapidly. Proactive up-skilling and retraining of people are critical. Companies are spending billions of dollars each year to develop courses, train their existing workforce, and onboard new hires. Many companies resort to mass training; the majority being inefficient web based and costly instructor lead training with some companies innovating in teaching and learning analytics to increase return on investment (ROI) for the many diverse learning and training interventions [1].

To match the pace of technological change and to scale up training, companies are leveraging online learning platforms and massive open online courses (MOOCs). In 2017, Class Central reported that 78 million learners took 9.4 thousand courses from 800+ universities [2]. MOOCS platforms have shifted from free to fee based certificates and corporate partnerships. The “freemium model,” where learners pay to get industry-validated certification is rapidly gaining popularity, especially for technical and professional learners [3]. The degree of coordination between learning platforms and corporations varies [4]; some companies encourage employees to pursue off-the-shelf courses that teach job relevant skills; while other companies have engaged educational providers to co-create courses and certificates that meet industry specific needs to help close the theory–practice gap [5–8]. Online courses and short topical certificates with proper instrumentation reduce costs, scale to a broad cohort of geographical dispersed learners, support (a)synchronous learning, and generate real-time, micro-level data from thousands of learners using different types and sequences of learning modules.

Recent advances in course instrumentation and advances in learning analytics and data mining make it feasible to use detailed clickstream data to understand and support online teaching and learning [9]. They make it possible to run in-vivo experiments on how people learn, which supports efforts to advance the goals of personalized learning, as outlined in the National Academy of Engineering grand challenge [10, 11]. Learning analytics and visualization make it possible to evaluate and compare content modules, teaching methods, social learning practices and course design at scale with the goal of optimizing learning outcomes and competencies based on goals of both individual learners and organizations [12]. Results support data-driven decision making by various stakeholders including: learners, teachers and course designers, learning scientists, and platform developers [9]. Learning analytics and visualizations combined promise rapid innovation for work-force development programs, where companies require a highly-educated “agile and adaptive workforce,” but to also give companies new opportunities to compute key learner competency, proficiency, adaptability to change, and social network capacities [13].

In the next section, we introduce learning analytics as applied to optimize workforce training and define units of analysis as well as models and metrics, and we review prior work on analytic models and visualizations of learner engagement, performance, and trajectories. In the data and methods section, discuss the edX data and data processing workflow used in our work. In the results section we present course structure and network visualizations that capture learner trajectories in online courses. The metrics and visualizations are exemplified and validated using events logs of activities by Boeing engineers taking the MITxPro Course “Architecture of Complex Systems” in Fall 2016. A discussion concludes the paper with a discussion of opportunities and challenges and planned future work.

## Learning analytics applied to online workforce training

Learning analytics (LA) refers to the measurement, modelling, and communication of learner data to understand and optimize teaching and learning and the socio-technical environment in which it occurs [14, 15]. With the rise of MOOCs and technical learning platforms (e.g., edX, Udacity, Pluralsight, Udemy, Lynda), applications in workforce training have increased, driving investments in LA applied to data generated by millions of learners taking online classes [16, 17]. LA uses diverse temporal, geospatial, topical, and network analyses and visualizations to answer when, where, what, and with whom questions [9]. LA provides predictions (e.g., of at risk learners, procrastination, and concept mastery), supports authentic learning (e.g., learning modules and paths that emerge based on authentic individual interest and engagement); increases our understanding of how people learn; and quantify return on evidence based instructional design that enables learners to explore and individual tailored and authentic learning paths.

LA applied to online workforce training poses a number of unique characteristics that must be considered when developing courses for this group. Three are listed here:

- Learners are domain experts that have responsibility for their own knowledge and learning experiences; however, it is important to recognize that technical, professional and managerial expertise and skills are distributed throughout the workplace;
- They prioritize practical transfer of knowledge to theoretical understanding. They are motivated by rapidly transferring course knowledge to workplace applications and are interested to gain skills that promote career advancement.
- Learners will generally vote with their feet if they do not see value in a course.

Consequently, workforce training and skill development differ from formal education in two important ways: 1) Learning interventions are typically short—a few hours, days, or weeks long—instead of many years of elementary, middle, high-school, or (under)graduate education; 2) Work performance before and after the intervention or course can be compared as the learner typically works the very same job before and after the intervention—which differs substantially from enrolling a high school learner and graduating him/her as an employee. Past efforts that evaluate workforce training show that learners have poor retention of information, they “forget” much of the content—if they cannot transfer knowledge to job demands [18–21]. Close collaboration between instructors and corporate experts is needed to design courses that truly make a difference.

Validation of workplace learning efforts makes it possible to address essential questions, including: what are the correlations between course resources and pedagogy with learner engagement and knowledge retention and translation, including activities beyond the course? What instruments can unlock and make visible knowledge that senior experts have so that it can be transferred to junior experts? What knowledge and expertise cut across organizational silos and should be taught to all experts—independent of department or seniority? How does one measure the impact of educational investments on an organization and individuals? What changed because of the course? What is the monetary or other return on investment (ROI) for a course?

The short durations of online courses make it possible to rapidly test, experiment and optimize workforce training efforts. Analysis of learner knowledge (prior and post-course), performance, and changes in work processes allow researchers to evaluate interventions by measuring an individual’s or groups’ growth and job performance as a measure of ROI on the investment in training. The work presented subsequently focuses on continuous experimentation, continuous improvement (of courses) guided by theory. Evaluation aims to improve course design and evaluation techniques; it analyzes data to isolate what is essential, distinguishes between special cases and what is systematic, and must understand variation to avoid chasing noise. Results support course optimization and development strategies for future training initiatives that reduce forgetting and improve productivity and worker satisfaction and retention.

While learning analytics research has been performed for over a decade, there does not exist an agreed-upon standard vocabulary of key units of measurement to describe and interlink the content, structure, and dynamics of courses. Similarly, current efforts to standardize data captured from different LMS or other services (e.g. Caliper and LTI standards). Yet, when developing a course, it is desirable to maximally exploit features and functionality offered by an LMS—if a course is purely online with no residential or other in-person component, nothing else but the LMS functionality can be used to engage and teach. Specifically, the LMS must support 1) content delivery (reading e-books, watching videos, taking exams) and 2) accessing data about what (consumption of LMs, production of exam results, social) activity each learner performed at what moment in time.

As for online courses, analytic and predictive models aimed at understanding how people learn or teach rely on data collected by LMS systems, because it captures instructor course design and knowledge structures, and instructor and learner activity logs. These data may be supplemented with demographic data, survey instruments and secondary analysis of course materials. The results of analysis and modeling must be communicated to all stakeholders working with the system to gain the most insight (e.g., translated into a language that key stakeholders understand).

Different stakeholders—instructors, course designers, learners, learning scientists, or corporate leadership—have different *roles* to play to make an online course or certificate successful; they also have rather different *insight needs*. Learners likely need a rather different presentation of analysis and model results than instructors; as both groups will need to act in different ways. Ideally, they work together to exploit synergies and leverage their diverse yet complementary expertise. Learners must be considered co-equal agents in a course, and should be made aware of the processes of their own learning and how their data will be used as to continuously improve course resources and the learning management system. Teachers (and over time, learners) are very familiar with the course structure and hence this structure makes a good ‘base map’ or reference system; typical learning pathways can then be rendered as data overlays. Model results and visualizations must be actionable, i.e., needed changes can be understood and implemented by key stakeholders. For example, it might not be possible for instructors to provide one-on-one teaching for learners due to time and budget constraints; however, they might be able to use the LMS system to encourage peer-support and to implement peer-grading.

In this paper, we distinguish actors and content data. *Actors* include learners, instructors, and designers. For each learner, there commonly exists data on demographics (e.g., age, gender, education, geolocation); timed learning module sequence; timed test/exam results recorded at the question level; and social interaction (e.g., in discussion forums, peer grading, or team projects). The latter data might be incomplete as learners might use email, phone call, zoom, or other means to collaborate. *Content Data* refers to two types of data: 1) data captured from LMSs or other platforms such as Course structure—a set of learning modules (or units) that may have different types (video, web site, exam), demographics and performance, event logs (see also section edX event logs); and 2) data provided by instructors, e.g., an estimation of time to be spent and cognitive load per module, suggested sequence of modules, information on what (sub)modules support what learning gains; what learning gains are tested by which exam questions.

Typical models can be categorized into those that capture temporal aspects (e.g., when is a learner or team actively consuming materials or producing exam or project results? Are there bursts of activity? Is activity increasing/decreasing over time?), geospatial (e.g., where do learners come from and what time zones are they in? Do geospatially close learners collaborate more often?), topical (e.g., what expertise do learners have before and after taking the course? What learning trajectory do they choose to gain what knowledge over time?), and network aspects (e.g., with whom do learners talk, work with and/or whose work do they follow and review? [9]. Typically, engagement (i.e., with content and/or social media) and performance (i.e., measured by quizzes, homework, and/or exams) are analyzed separately. However, it is often insightful to see the authentic patterns of interactions learners make across these course resources.

Communicating the results of analytic and predictive models in an effective way to different stakeholders is non-trivial. The statistical features that have the strongest impact prediction might not be the features that instructors or designers can manipulate or control. Most learners, instructors, or corporate leadership are unlikely to gain actionable insights from looking at the output of Hidden Markov Models, random forests algorithms, or Support Vector Machine hyperplanes without well-designed visualizations that account for individual stakeholders insight need and intended use cases.

The visualization work presented in this paper uses a modified state transition networks to analyze and visualize learner trajectory. Learner trajectory networks reveal how individual or groups of learners use and traverse course structures and materials over time. Early work to visualize learner transition between types of resources used as state transition networks that were based on learners’ education software audit trails or clickstream data from logs. Similar state-transition dyad networks have been created to visualize the use and transitions of course materials in the context of problem solving activities (e.g., homework or exam) based on edX clickstream data for all learners in a course [22]. The networks use a two column horizontal layout, where nodes represent types of content modules found in edX courses and are sized by the total time spent by learners over the length of the course; edges represent the transition from left to right, and are sized by the proportion of learners in the course taking a path. The networks reveal the types of resources learners rely on when studying exams and completing assignments. For the course analyzed, activity and assessment module ego-networks represent learner’s patterns of engagement by visualizing use of transition of other course resources; in the course analyzed, networks reveal that a large proportion of learners’ transition to discussion forums after homework activities; while learners spent more time with videos than other types of resources after visiting course exams.

Kizilcec, Piech and Schneider [23] explore disengagement in MOOCs by classifying learners based on their engagement with assessments and clustering these state assignment to characterize high level patterns of learner engagement across different structures and instructional approaches for three computer science courses. For each module of the course, learners were assigned one of four classes based on their assignment submission status for the period: on-track, behind, auditing (no submission for the period), and out of the course (inactive in period). A state transition network is used to visualize the flow of learners between engagement categories throughout each module of a course. Node size represents the number of learners assigned to a class, weighted edges are directed left to right and show the proportion of learners from one class transition to another class in the next module. Results of the study show that MOOC designers are not considering alternative types of engagement when developing courses, with current designs centered on traditional models of learner engagement.

Coffrin, Corrin, de Barba [24] use iterative analytic methods to analyze online courses and develop a heuristic to classify learners into subpopulations and to use clickstream data to create state-transition diagrams of movement between modules and resources in the course. Three cohorts (qualified, active, and auditing) were identified based on learner grade performance within the first weeks of the course. To understand learner conformity to the linear sequence structure of a course and impact of course design, state transition diagrams visualize cohorts’ use and transitions between sets of video and sets of quizzes and exams that are topically linked. The state diagrams use a linear layout to sequence the nodes, which are sized by the unique number of learners participating on a given topic or set of resources. Transitions represent directed movement in the network: arcs above nodes represent movement “left to right” in the course content; arc below nodes show “right to left” movement; perpendicular edges represent proportion of learners disengaging from the course. Visualizations revealed similarities in engagement patterns for auditing learners across courses; qualified learners have more transitions between videos compared to auditors; and open course designs show more transitions between quizzes and videos than course that gate access to content.

Time-graphs, weighted directed networks, have been created to reveal the topology of course content that emerges from the collective click stream activity of learners’ data in small online courses [25, 26]. For this study, time-graph nodes represent course content and are sized by frequency of visits by learners; edges represent transitions between content, and are weighted by the average duration taken by learners between moves. The analysis of the pathways reveals learners adherence to course designs, as well as distinct patterns in the time taken to proceed between resources of the same types (e.g. transitions between course assignments are longer than transitions between lectures), and reveals breaks taken by learners between lectures and content. Time-graphs of learner use of materials could support recommendation engines for personalized learning in online course by modeling both engagement and temporal delays between transitions that emerge in course content for identified groups and individual learners’ activity.

Davis, Chen, Hauff [27] take a process mining approach to analyze clickstream logs from four edX courses to evaluate learners adherence to the designed course pathways found in MOOCs using discrete-time Markov chains for interactions and transitions across video watching, and between eight types of event logs extracted from these courses. Three trajectory visualizations were presented, the first was used to highlight the general designed paths for the courses that range from simple linear path to multiple cycles through videos, quizzes, progress checks and forums. Nodes represent the event states in the logs and multiple edges represent the steps designed. The second trajectory network visualizes the patterns of transitions across videos in the first three weeks in each course, for passing and non-passing learners. Using visualization analogous to [23, 24], show that passing learners are less likely to deviate from the designed path and will skip intro videos; both groups are more likely to move forward than backwards in content. The finals set of visualizations show the results of the Markov chain analysis and shows transition probabilities between the types centered in the analysis; edges and nodes are colored based on one of eight event types analyzed, and reveal that instructional design can impact transition probabilities across courses and grade performance groups, and that MOOC platforms do not have way to expose non-designed paths for learners that might aid learners in completing a course.

Learner trajectory analytics results have revealed patterns of engagements through data mining, machine learning and visual analytics for different groups of students based on engagement and certificate status and grades, which can be used to improve reporting of course outcomes based on classification and clustering of students’ clickstreams [23, 24]. These networks also highlight common pathways taken by groups and reveal the ways that current instructional designs and practices do not account for how learners use these resources to acquire and build knowledge in real-time. The work taken on in this project builds on these methods, metrics and visualizations to explore overall patterns of learner engagement with different types of content modules and individual interaction patterns to identify subpopulations within online large professional development courses in addition to certificate status and grade performance.

## Data and methods

The studies described above use a variety of measures and metrics to analyze learner engagement and transitions in online courses. Some common themes emerge in the metrics and variables used in these studies that can be broken into module use (node) and transition (edge) measures. Module measures include: total number of events [22] and dwell times for using modules [22, 25], total number of learners active or participating in a module [22–26]. Transition measures include the proportion of learners transitioning between modules [22–24, 27] and the time between transitions [25–27] and designed course paths and state transition probabilities [27]. Likewise, these studies have classified data for different types of engagement, including assignment submissions [23], grade performance after first two weeks [24], and certificate status for courses [27].

Building on and extending the units of measurement, metrics, and visualizations introduced above, we analyzed data from a MITxPro Course “Architecture of Complex Systems” delivered via the edX platform in Fall 2016. A total of 1,611 Boeing engineers registered and signed an informed consent waiver acknowledging that their data can be used for research. Of these, 1,565 engineers were active in the course generating nearly 31 million click event records while accessing videos, projects, and assessments. Some learners generated over 100,000 separate events. All but 255 engineers passed the course, resulting in a completion rate of 84.1% that is much higher than achieved in a typical MOOC. Only 22 engineers failed to meet with 70% performance threshold and had to repay the $1,100 course enrolment fee to Boeing; the remaining learners’ either dropped or completed the course during another run. Note that engineers are a unique in terms of extensive technical expertise and a rather unique drive to accomplish perfect scores, keen interest to be efficient when learning, and are highly motivated to complete courses because of monetary incentives. Subsequently, we detail edX data, analysis, and visualizations of learning trajectories.

### EdX data

Data from an edX course comprises a (1) course database, which captures **course structure**, and **learner demographics and performance data**; and (2) daily **event logs**. All three data types are briefly described here; details can be found in the edX documentation [28].

#### EdX course database

*Course Structure*. The hierarchical structure of learning modules exists in JSON format. Modules can be of different types such as html, problem, video, or discussion. The five-level JSON tree hierarchy and associated sequence of learning modules, and together with a one-dimensional base map of course modules organized linearly from beginning (left) to end (right) according to instructor design. The root node provides course metadata; second level corresponds to course chapters that outline the major grouping of content; third and fourth levels represent learning modules; fifth level nodes are course content blocks (e.g., videos, html pages, problems, assessments, etc.). Module identifiers from various levels of the course structure are linked to the course clickstream event logs (see Data Types and Descriptions) through a variety of references specific to the type of module and action recorded.

*Learner Demographic and Performance Data*. Learner data includes learner generated artifacts, system and social interactions and learning outcomes are captured in a SQL database. Among others, the data captures first and last learning module states, final course grade, certificate status, discussion forums posts, and team data. Discussion activity for the course was supported and captured via Piazza forums but cannot be linked to other activity data and hence is not used in this study.

#### EdX event logs

*Event Logs*. Learners’ interactions (but also course developers’ and instructors’) in a course are emitted as browser and mobile events, and the LMS responses to user interactions are emitted as server events; both are available in real-time as streaming JSON (ndjson) records. Logs of learner activity are captured and shared a daily for each edX course. The “Architecture of Complex System” logs contain events associated with content knowledge (videos and html-text modules), ungraded problems (e.g., multiple choice, drag-and-drop, word cloud problems), and team-based open assessments, see supplemental materials for details.

### Data processing workflow

**Fig 1:**
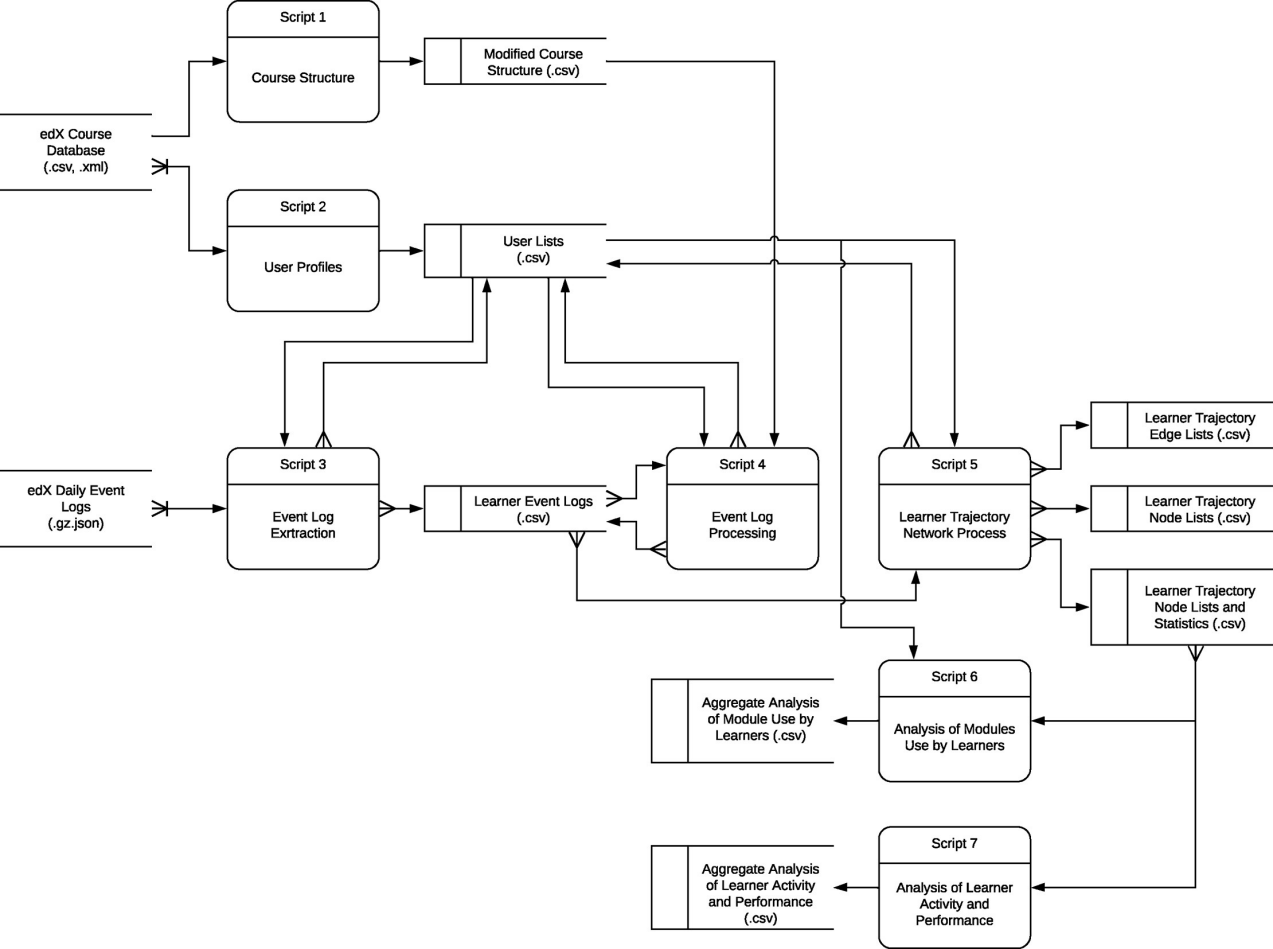
Data flow diagram shows the processing steps used with edX course data.

Data processing, analysis and visualization were completed using R statistical software. Course data and event logs were processed to study course structure, learner’s use of course content modules, and learner’s performance, interaction and trajectory with course content in a workflow (Fig 1). S1 Supporting information provides a description of the R data processing workflow, a link to the GitHub repository, and notes on using the pipeline, data reporting and other supplemental materials. S1 Appendix provides data dictionary for the outputs generated by each script that is apart of the data processing workflow, and include descriptions of data fields and information describing how fields were calculated; S1 Dataset provides five sample datasets of data processing results; individual data files are referenced below. Analysis and visualizations were implemented to support the study and optimization of other courses delivered via the edX learning management system.

The UML diagram shows the flow of data edX course data through the R processing scripts that were implemented to generate data for statistical and learner trajectory network visualizations. The initial first two scripts in the data processing workflow are used to extract the course structure and to identify learners who are enrolled in the course. Using a third script, daily event logs are processed to create a unique event logs for each enrolled learner, which are then separated into groups: active learners, and learners with no activity or who only interact with a course’s non-content modules. The fourth script processes active learner logs to order events, calculate event durations, remove redundant events from the logs, and align all events to the lowest level of the course hierarchy. The final script creates a learner trajectory network for each active learner in the course (see script documentation for details).

The edX course structure contains references to modules used for course development, testing, and overcoming short-term technical issues (e.g., pages that the instructors used for learner team sign up). These modules were not used to teach the final course and excluded from this analysis resulting in 310 remaining content modules. Next, with *Script 4*, we process individual learner event logs to identify events associated with a set of 16 event types that capture learners’ interactions with course content, assessments, and course navigation. These event types are learner initiated actions (i.e. browser events), with the exception of some problem modules events, where a server event captures more the accuracy of student responses to a problem question. Unlike Davis, Chen, Hauff [29], we do not transcribe events to a custom data scheme of event types; rather, native edX event types are used. In line with Geigle and Zhai [30], we identified each session as the sequence of events by a user (or a server response to a user action) broken at gaps greater or equal to a 60-minute break between events. Most learners will have multiple sessions. Each of these sessions contains a sequence of events from the event logs. Events that have over 60 minutes of a dwell time are replaced with averages event times for modules of the same type for a given learner to avoid over estimation of learner time on a given module.

#### Learner course activity and performance measurements

Only enrolled learners were retained in the dataset (i.e., instructional team members or staff were excluded), all have confirmed enrollment in the course, and perform some learning activity in the course by accessing multiple course modules (e.g., spending time watching a video or answering a conceptual problem). Measurements of a learner’s interactions with individual content modules are calculated *Script 5* based on an individual’s processed log files to create a learner trajectory network. *Script 7* reads learner trajectory node data to create aggregate statistics describing individual’s overall course engagement and performance, which include: the number of unique sessions per user; number of unique days active in the course; number of unique modules access overall, as well as videos, problems and open assessments; total number of events and total time in the course and for select actions types (e.g. event associated with video, problem, open assessments, peer review, and transition activities); the number of problem attempts, total correct responses and total points earned

#### Content module activity measurements

Analysis of learner interactions with content modules performed by *Script 6*. The script analyzes each student’s learning trajectory network node list to create a set of descriptive usage statistics for each content and assessment modules in the course. The analysis of content modules provides an exploratory analysis for how different types of modules are used by learners. Analysis was performed using all active learners enrolled in the MITxPro Course “Architecture of Complex Systems”; however, this analysis could be applied to sub-populations or groups identified by instructors or researchers that are of interest for comparative analysis. Insights gained from analysis of aggregate use of content modules is important for instructors and instructional designer to understand what and how content is used across the course overall or subsets of learners for post-hoc evaluation and to identify potential interventions or modifications to the course in future runs. This type of analysis also helps researchers generate hypotheses and experiment designs, in selection of appropriate data analytics methods and normalization procedures; as well as the design and application of machine learning models for personalized learning.

## Results

### Course structure and learning trajectories visualizations

The course structure tree and learner trajectory networks were visualized using Gephi. Visualization of the multivariate analysis were generated in R using the ggplot2 library.

#### Course structure tree

**Fig 2:**
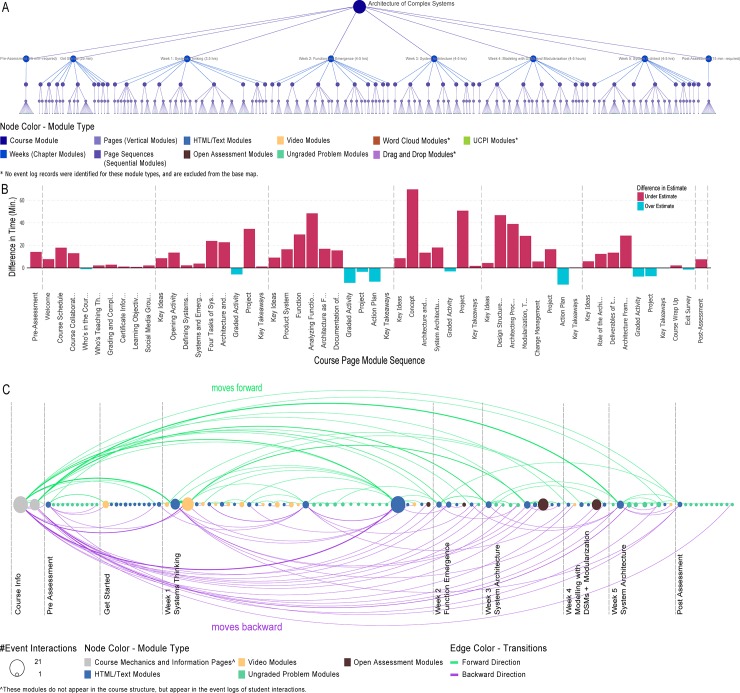
EdX course structure provide levels of learner interaction analysis and guide base map construction. (A) Shows the 5-level course module hierarchy used in the “Architecture of Complex Systems” course; (B) a bar graph shows difference between instructor’s predictions and the average time learners spent on the same set of modules, aggregated at the page sequence level of the course hierarchy; (C) learner path shows temporal sequence of learning modules accessed by a high performing learner.

Visualization of the course structure reveals the nested hierarchical organization, the composition of course materials, activities and assessments, and temporal ordering of learning modules of the course, see Fig 2A, Data A in S1 Dataset. The leaf nodes of the hierarchical tree represent learning modules that learners are accessing in sequence to learn. The course structure provides a means to aggregate learner interactions at different levels of granularity and to construct base maps to overlay learner interaction data and trajectories, see Fig 2B and 2C.

#### Instructor’s temporal predictions

Course instructors estimated the time it would take learners to complete content and activity modules in the course. Fig 2B shows the difference between instructor’s estimates for course materials and the average time taken by learners in the course for a given content; Fig 2B was generated based on aggregated analysis of Data A and C in S1 Dataset. As can be seen, for the “Architecture of Complex Systems” course, instructors often under estimated (in red) the amount of time an average learner would need to complete content and activity modules, except for group assignments where time was overestimated (blue). The visualization also shows differences in time spent by learners per week—time demands increase through the third week of the course but reduce during the last two weeks of the course.

#### Learning trajectories

A learner’s complete learning trajectory can be extracted from their event log data and the edX course structure, see Fig 1. Learning module are ordered based on instructional design from left (first) to right (last), with dividing lines for pre and post assessments and weekly module groups. The type of different content module types is encoded using color: videos (yellow), HTML and text modules (blue), and ungraded problem modules (green). Learner interactions with course content modules were aggregated to nodes in the network; learner pathways show transition sequences between content modules in the course and are represented by arcs, see Fig 2C, and exemplary Data B in S1 Dataset. Green arcs indicate forward jumps while purple arcs denote jumps to earlier modules.

**Fig 3:**
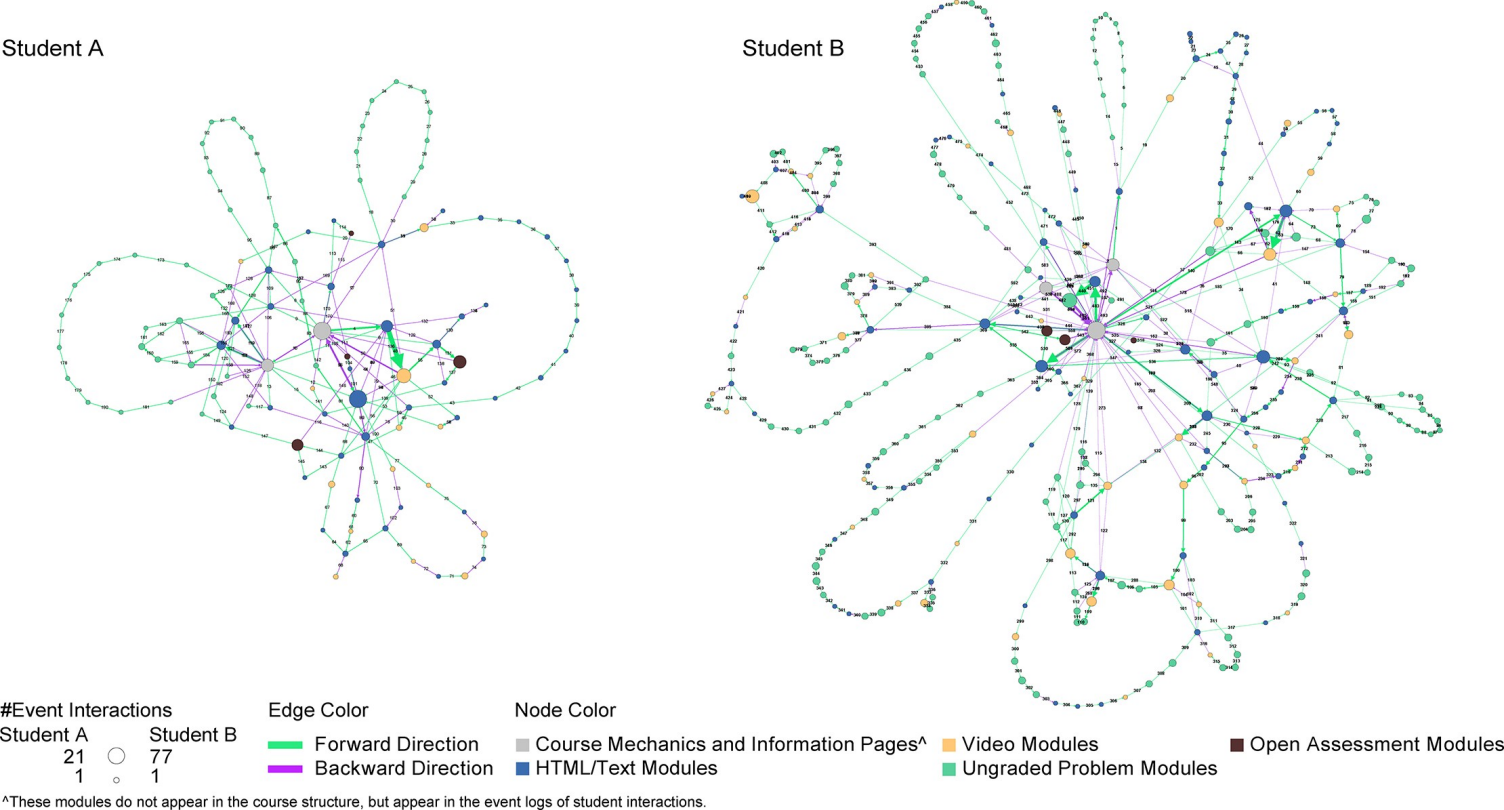
Learners’ path overlaid on force-directed layout of used course modules. Learner path of a learners with a high (left) and a low (right) performance scores overlaid on force-directed layout of course modules.

An alternative layout is shown in Fig 3, where the two learner paths are rendered using a force-directed layout. While Learner A on left with a 99% passing grade uses fewer transitions (229 transitions over 103 edges), s/he spends much time on content pages, video, and group problems (total of 208 minutes). Learner B on right achieved a 71% grade; s/he engaged in many more transitions (584 transitions over 431 edges) and spent much time on course mechanics (navigation/portal) pages (total of 277 minutes). Visualizations of learner trajectories from event logs provide a lens into the study and navigation strategies employed by learners in a course. Comparing the two learners in Fig 3 shows that Learner A uses the html pages and videos during the first weeks of the course, and then shifts to using the problem and video modules in later weeks; while Learner B used each type of module throughout the course. Both learners show similar number of interactions with accessed modules over html, video, and assessment modules; on average, Learner B used 3.63 events per problem, while Learner A created 1.43 events per learning module.

### Multivariate analysis and visualization of learner interactions and performance

**Fig 4:**
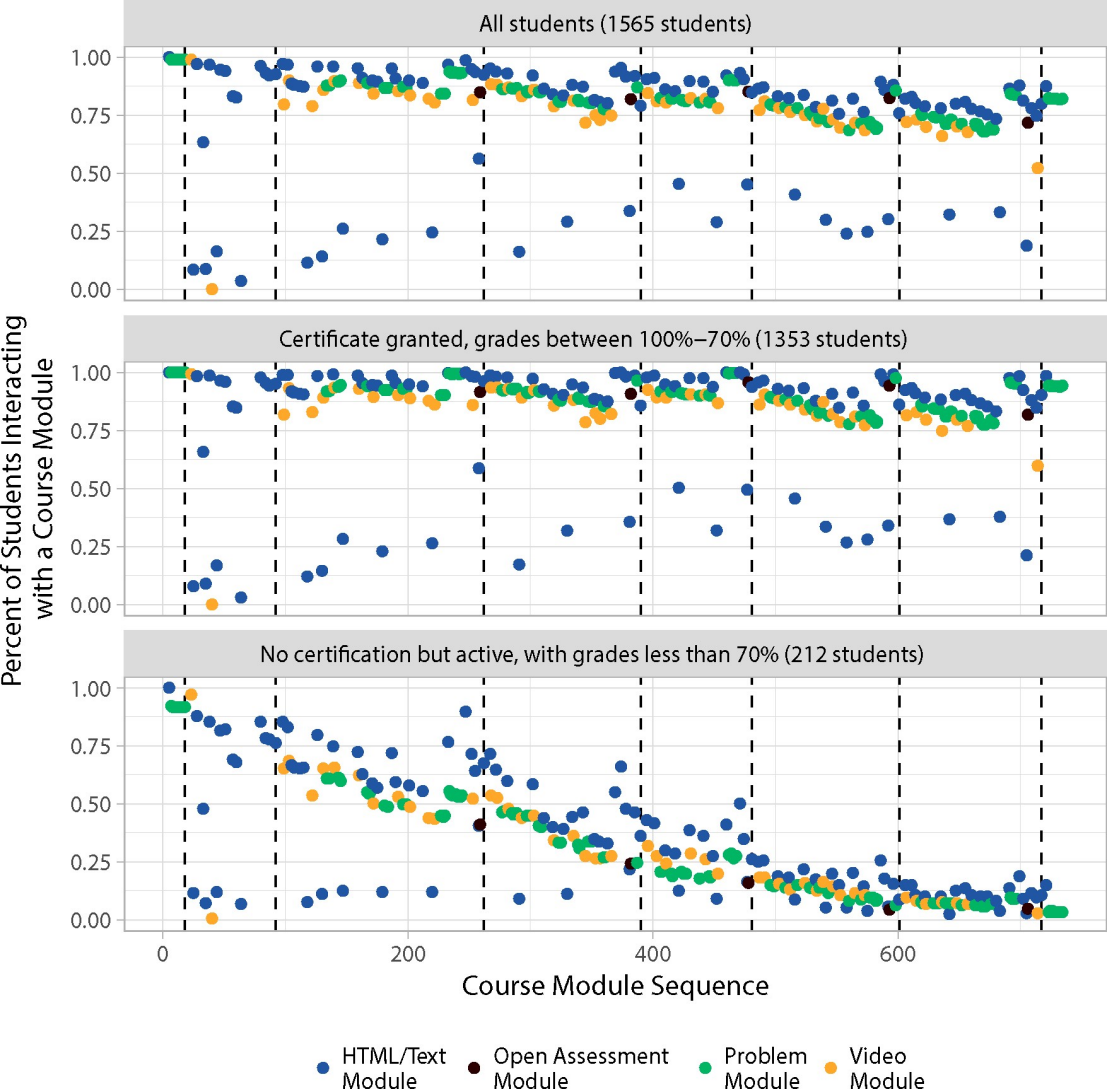
Percentage of learners accessing a module in course sequence. Plotted are the 310 of 551 modules at lowest level of course structure used by learners. Vertical lines indicate the last module of a given course section.

Performance data for all learners that interacted with a content module, as well as learners earning a course certificate (grades 100%– 70%), and failing learners (grades < 70%) are graphed in Fig 4 based on Data D in S1 Dataset. As in Fig 2, learning modules are plotted from left (first) to right (last) with dividing lines for pre and post-tests but also week 1–5 course sections. A visual comparison of learner behavior in week one shows a difference in use of course materials in the course pre-test and first week of the course, with disengaging learners interacting with fewer problems and videos in the course compared to the learners earning a certificate.

**Fig 5:**
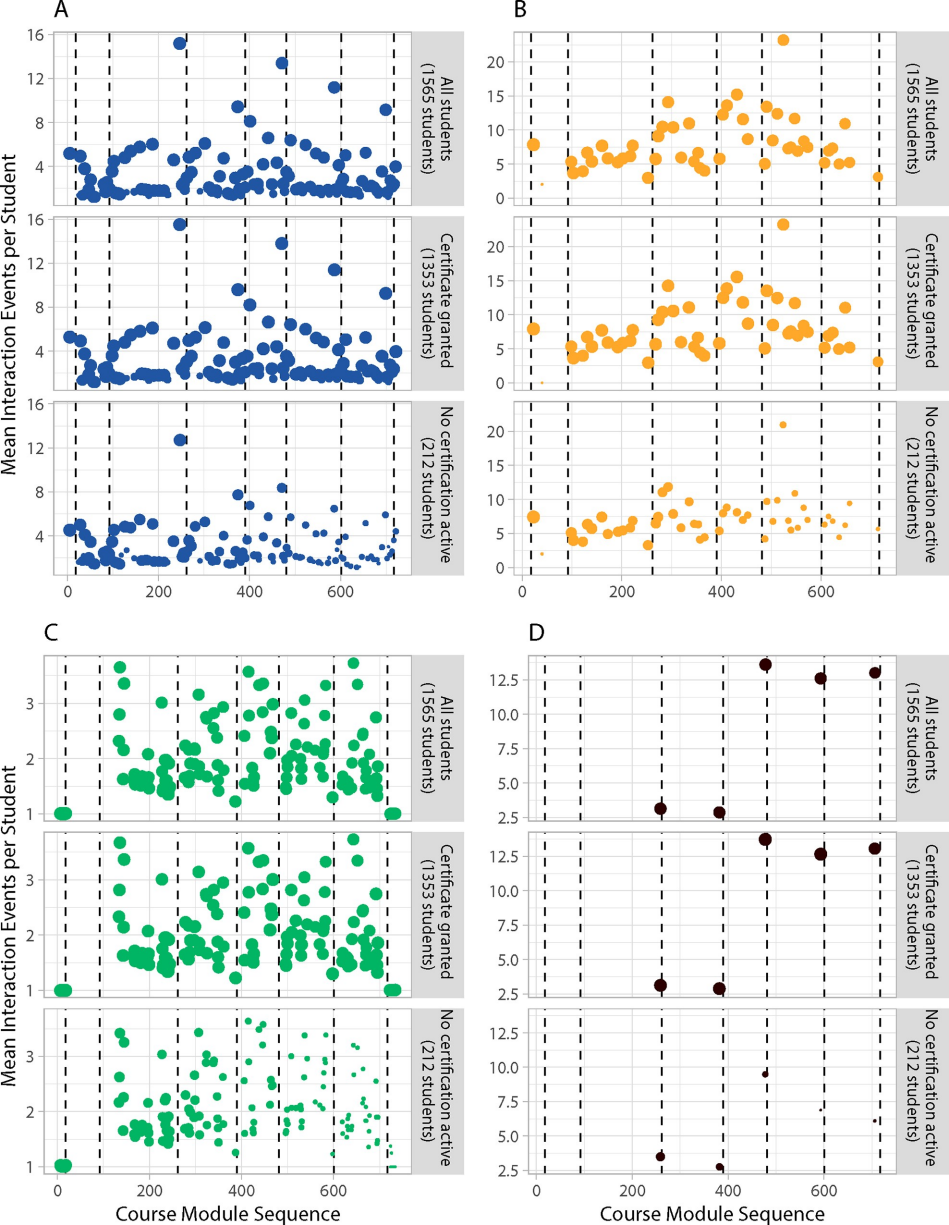
Mean number of interaction events per learners with a given module in a course. (A) Plots the 117 HTML modules, (B) shows 49 video modules, (C) 139 ungraded problem modules, and (D) 5 open assessment modules from the lowest level of course structure that were used by learners. Vertical dashed lines indicate the last module of a given course section. Circle area size is linearly scaled to show the percentage of a group that interacted with a module.

Scatter plots in Fig 5 show the mean number of interactions learners have with different module types, and is based on Data E in S1 Dataset. The size of the module shows the overall percentage of learners from that group that interacted with the module. Comparison of the overall interaction data reveals distinct use patterns that depend on module type and the ability to capture or infer module use by learners. Problem and video modules show major differences in the number interactions; while open assessment modules show an increase in use over the duration of the course. Use of HTML/Text modules shows a log distribution of use, which is likely caused by the inability to show direct use of these modules by learners; instead use of HTML modules is inferred by processing learner logs when extracting learner transitions networks.

**Fig 6:**
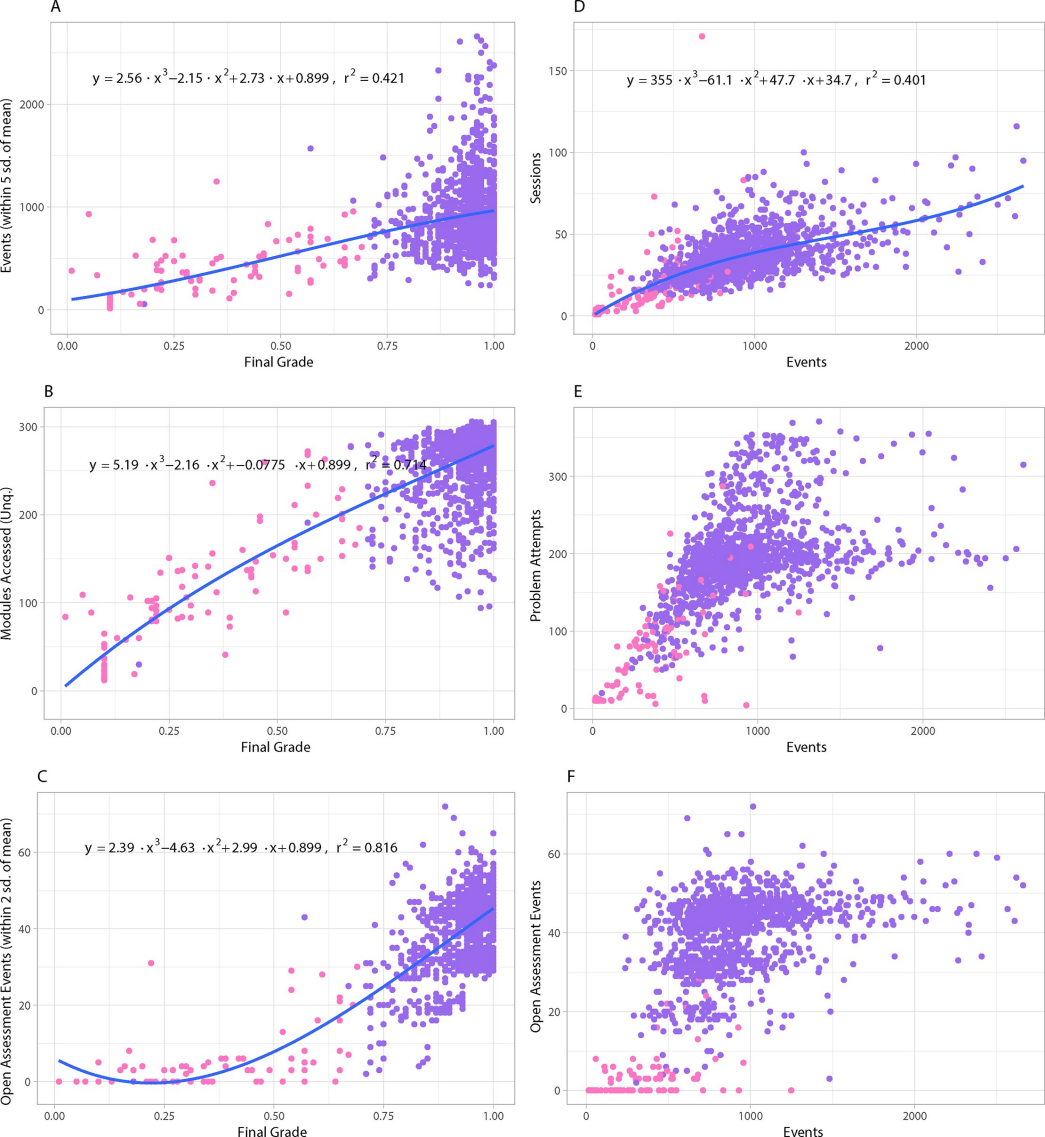
**Correlation of different variables with final grade (left) and number of events (right).** The plots compare: (A) grade and events, (B) grade and unique modules accessed in the course, (C) grade and open assessment events, (D) events to cumulative user sessions, (E) events and problem attempts, (F) events and open assessment events.

Scatter plots of the relationships between final grade and cumulative interaction events in a course and linear models are depicted in Fig 6, based on Data D in S1 Dataset. Generalized features, such as the cumulative count of events and sessions, tend to provide moderate correlations to final grades and each other. However, features related to specific activities show distinct patterns of behavior of course material interactions. For example, sparse use of open assessment modules and peer-feedback model distinct clusters of learners based on participation and engagement within the course, see Fig 6C and 6D. Comparing learners’ interaction with problem modules and their cumulative interaction events shows an interesting pattern of learner engagement (Fig 6E). A majority of learners fall into a distinct range of total problem interactions (between 100 to 225 interactions), which reflects the total amount of problem modules in the course. However, echo signal is also present in the graph, which is caused by a subset of learners who on average submitted more than 1 responses per problem module, when compared to other learners in the course.

## Discussion

Study results presented here showcase the value of visualizations for examining and communicating learners’ engagement, performance, and trajectories in online courses. The data analysis and visualization methods were exemplified using an extensive corporate dataset of 1,608 Boeing engineers. Visualization results show course structures and patterns of learner interactions with course material, activities, and assessments. They can be used to optimize the sequence and content of online course materials, to inform learners and instructors about learning progress. Novel learner trajectory visualizations make it possible to understand and communicate how individual learners access course content modules, revealing patterns of learner engagement, content access strategies, social interactions, and performance. They aim to empower instructors and corporate partners to optimize current courses and to develop more efficient future courses.

The presented study helped identify areas where instructors and course designers can improve course instrumentation to help capture data used in actionable analyses and visualizations. Subsequently, we list major issues that should be addressed by instructors, user logging, and overall course design to further improve online workforce training and associated LA.

### Instructors

*Time estimates*. Instructor provided course time estimates for a chapter module do not necessarily align with time estimates associated with pages of low-level edX blocks. *Task Difficulty*. Ideally, instructors provide estimates for cognitive load (e.g., using Bloom’s taxonomy typology) for each learning module based on test runs and taking into account learners’ expertise. These time estimates should be compared with time spent data collected for different learners (cohorts) and adjustments should be made as needed. *Sequence of Learning Modules*. What is the instructor prescribed sequence of learning modules (for different learner cohorts) and how does it differ from actual trajectories different learner (cohorts) take? *Consumption-Production Matrix*. What content is tested in which problems, exams? Is there any content that is not tested or are there tasks/skills that are tested but not taught? *Success Metrics*. Final grade may not be the best indicator of learning success. What other indicators count?

### User logging

*Unique User ID*. MOOCs that use different LMSs, social platforms (and additional services for teamwork) must robustly link the diverse datasets (e.g., via learner IDs) so that learner engagement, performance, and social interaction can be studied. *Distinguishing Types of User*. Actions made by the instructional and course design teams differ from learner activity. Both might be studied but need to be distinguishable. *Estimate Task Times*. It matters how long a learner takes to read a text, talk with others, and work on a homework or exam. Time per task must be measurable. *Unique Session and Module IDs*. Both identifiers must be captured consistently. In some MOOC platforms, session identifiers are captured for activities taken by a learner (i.e., browser events) but not for events generated by the system (i.e., server events). The latter must be computed situationally and under assumptions for server events, which can lead to errors in LA calculations.

### Course designers

*Video length*. How long is the runtime for each video? Is it ok if learners speed videos up and is this captured in log files? When do learners pause, rewind, and stop the video? *Assessments*. Were learners allowed to take the pre or post assessments and/or other assessments multiple times?

Given improvements in data, novel analytic models and visualizations of learning can be developed that capture key structural and dynamical aspects of learner’s trajectories at ever higher levels of detail and in a manner that is truly actionable for learners, teachers, and corporate partners. Ultimately, companies and other intuitions are interested to deliver high-quality, effective online education that keeps up with the accelerating pace of science and technology developments. They are interested to design courses with highly automated data collection that provides key insights in real-time; the integration of teaching and learning with the ever more digital nature of the modern workplace; continuous experimentation and improvement of workforce training; a stronger emphasis on social learning; and more effective means to transfer the extensive expertise of senior experts to the next generation of their workforce.

## Supporting information

S1 Supporting Information Learner trajectory network processing pipeline. Supporting information describes the Learner Trajectory Network Processing Pipeline used for analysis and visualizations introduced in this paper, and provides links to related protocol and GitHub repository.
(DOCX)

S1 Appendix Data dictionary for learner trajectory network processing pipeline. Appendix provides a data dictionary for the outputs generated by each script of the processing pipeline.
(DOCX)

S1 Dataset Sample data outputs of learner trajectory network processing pipeline. Dataset includes five files that were generated by the data processing pipeline based on course and student data from the MITxPro course, Architecture of Complex Systems, Fall 2016.
(ZIP)
