# Supplementary material for: Visualizing learner engagement, performance, and trajectories to evaluate and optimize online course design
Source: PLoS One. 2019 May 6;14(5):e0215964. doi: 10.1371/journal.pone.0215964 (PMC6502341; doi:10.1371/journal.pone.0215964)
Supplement: S1 Supporting Information — Supporting information describes the Learner Trajectory Network Processing Pipeline used for analysis and visualizations introduced in this paper, and provides links to related protocol and GitHub repository. (DOCX) [file pone.0215964.s001.docx]

**Supporting information**

**Learner trajectory network processing pipeline**

The edX Learner and Course Analytics and Visualization Pipeline is an R script processing pipeline used to with course and student level data from an edX course database or edX Data Package. The pipeline was designed to:

- extract and process course structures, learners’ event logs, demographic and performance data;
- create learner trajectory networks (see [Visualizations of learning trajectories in online courses (cns-iu/learning-trajectories)](https://github.com/cns-iu/learning-trajectories) for example visualization implementation);
- analyze the learner use of course content modules; and
- analyze overall performance and interaction measurements for an edX course.

Scripts are available for review and use as part of a project GitHub repository, available at: <https://github.com/cns-iu/edx-learnertrajectorynetpipeline>.

**Protocol**

A generalize protocol is available at Protocols.io, [edX Learner and Course Analytics and Visualization Pipeline, V.3](https://www.protocols.io/view/edx-learner-and-course-analytics-and-visualization-zckf2uw) [dx.doi.org/10.17504/protocols.io.zfhf3j6](https://github.com/cns-iu/edx-learnertrajectorynetpipeline/blob/master/dx.doi.org/10.17504/protocols.io.zfhf3j6).

**Data processing and analysis script descriptions**

Each script in the data processing and Analysis pipeline is briefly described below:

1. [edX-1-courseStructureMeta.R](https://github.com/mginda/edx-learnertrajectorynetpipeline/blob/master/edX-1-courseStructureMeta.R) script extracts a the course structure from the edX Data Package files. The course structure is used in processing log files and creating the node lists in learner trajectory networks.
2. [edX-2-studentUserList.R](https://github.com/mginda/edx-learnertrajectorynetpipeline/blob/master/edX-2-studentUserList.R) script processes user profile datasets from the edX Data Package to identify active students in the course, and exclude instructors, teaching assistants and beta testers from the user log datasets. The script generates a list of learners’ edX user IDs.
3. [edX-3-eventLogExtractor.R](https://github.com/mginda/edx-learnertrajectorynetpipeline/blob/master/edX-3-eventLogExtractor.R) script processes the daily edX course’s event tracking logs (which use streaming JSON format) for active students in the course. Logs are collected for each day of the course, combining all students’ actions in one file. The script loops through the known learner user identifiers generated by the *edX-2-studentUserList.R* to extract a raw event log for each student in the course. The logs are saved as individual CSV files. The processing speed of this script will be based on the number of students and their volume of recorded activity.
4. [edX-4-eventLogFormatter.R](https://github.com/mginda/edx-learnertrajectorynetpipeline/blob/master/edX-4-eventLogFormatter.R) script processes the individual students event logs, extracted by the *edX-3-eventLogExtractor.R* script. The script uses the course structure dataset generated by edX-1-courseStructureMeta.R script as part of the log processing. The script allows a researcher to identify the types of events that are maintained in the final event logs for a student for analysis. All events in the log are aligned to the lowest level of the course structural hierarchy; provides temporal ordering and event period calculations and outlier estimates. The script loops through the identified list of learners, and sorts students into further groups based on the size of their processed log files (for example, the script separates students with fewer than 10 events to remove them from the analysis). The script creates of two new lists of student users based on analysis of the processed event logs: active and inactive students who were not excluded by *edX-2-studentUserList.R* script.
5. [edX-5-learnerTrajectoryNet.R](https://github.com/mginda/edx-learnertrajectorynetpipeline/blob/master/edX-5-learnerTrajectoryNet.R) script creates a learner trajectory network for each student in the course based on the individual’s processed event logs and user list generated by *edX-4-eventLogFormatter.R*. The script first creates an edge list to document transitions between modules in a course, and then creates a node list that describe a student’s interaction with each low level module in the course. The script exports a node and an edge lists for each student as: 1) two CSV files, and as well as 2) a JSON formatted learner trajectory network that combines the nodes and edge lists datasets are combined into a single file.
6. [edX-6-moduleUseAnalysis.R](https://github.com/mginda/edx-learnertrajectorynetpipeline/blob/master/edX-6-moduleUseAnalysis.R) script uses the node lists generated by edX-5-learnerTrajectoryNet.R and lists of student IDs generated by edX-4-eventLogFormatter.R. The script aggregates the node lists from individual students’ learner trajectory networks to provide an analysis of overall student interactions and activity and course modules. Analysis is completed for modules at the lowest level of the course hierarchy. The results are saved as a CSV data that can be joined to the course structure dataset produced by edX-1-courseStructureMeta.R script.
7. [edX-7-studentFeatureExtraction.R](https://github.com/mginda/edx-learnertrajectorynetpipeline/blob/master/edX-7-studentFeatureExtraction.R) script uses the course metadata generated by the *edX-1-learnerTrajectoryNet.R* script, and output user list and processed student logs and list of student IDs generated by the *edx-4-eventLogFormatter.R* script. The script loops through the list of student processed event logs to create a set of frequency statistics of student activity in an EdX course (e.g. number of sessions, events, unique modules, event_types), calculations of temporal use of content (overall, and relevant module and event types).

**Sample data and visualization reproductions**

Sample data and documentation for visualizations created with R have been reproduced in a set of R Markdown documents that are available in the project’s GitHub.io page: <https://cns-iu.github.io/edx-learnertrajectorynetpipeline/index.html>

**A note on using the pipeline**

The processing scripts are provided under Apache License 2.0. Contributors provide permission for commercial use, modification, distribution, patent use, and private use. Licensed works, modifications, and larger works may be distributed under different terms and without source code. The scripts are provided with a limited liability and warranty; use these data processing scripts at your own discretion, and make preservation copies of any source data prior to use.

Additional modifications are likely needed to make use of this pipeline when processing other course datasets that use the edX Data Package format specification. Organizational implementation of the edX learning management systems may use customized event log tracking systems, courses may use different types of edX block modules, and logs may include types of events that were not encountered in this project (e.g. error events, or edX discussion forums). An exploratory analysis of the course structure and event logs is advisable at the outset of a project.
