# Supplementary material for: Visualizing learner engagement, performance, and trajectories to evaluate and optimize online course design
Source: PLoS One. 2019 May 6;14(5):e0215964. doi: 10.1371/journal.pone.0215964 (PMC6502341; doi:10.1371/journal.pone.0215964)
Supplement: S1 Appendix — Appendix provides a data dictionary for the outputs generated by each script of the processing pipeline. (DOCX) [file pone.0215964.s002.docx]

**Appendix**

**Data dictionary for learner trajectory network processing pipeline**

**edX-1-courseStructureMeta.R data processing outputs**

The first script in the pipeline extracts a dataset that describes the course modules and structure for an edX course based on a course *{org}-{course identifier}-{term}-course_structure-prod-analytics.json* file. The script creates two data tables: 1) course metadata, 2) a modified course structure.

Table 1 describes the course metadata file of the course name, course identifier, and start and end dates for the enrollment and period the course was offered.

Table 2 describes the course structure created by the script, which is ordered by the identified module sequence; the course structure is used by multiple scripts in the data processing pipeline, see Data A (*dataA.csv*) in S3 Dataset.

**Table 1. Field list for {org}-{course identifier}-{term}-meta.csv (*dataA.csv*).**

| **Field Name** | **Description** | **Data Type** |
| --- | --- | --- |
| id | Course identifier for edX system; format: {org}+{course identifier}+{term} | String |
| metadata. display_name | Course name displayed in edX system. | String |
| category | Identifies the category of edX course module; in this case, the field should always be “course.” | String |
| metadata.start | Datetime for when course starts.  Format: YYYY-MM-DD hh:mm:ss | Datetime |
| metadata.end | Datetime for when course ends.  Format: YYYY-MM-DD hh:mm:ss | Datetime |
| metadata. enrollment_start | Datetime for when course enrollment starts.  Format: YYYY-MM-DD hh:mm:ss | Datetime |
| metadata. enrollment_end | Datetime for when course enrollment ends.  Format: YYYY-MM-DD hh:mm:ss | Datetime |

**Table 2. Field list for {org}-{course identifier}-{term}-module-lookup.csv.**

| **Field Name** | **Description** | **Data Type** |
| --- | --- | --- |
| id | This field is the full edX course module identifier that incorporates the course identifier, module type/category, and edX module identifier, which is typically a 32 character alphanumeric unique identifier string, except in the case of the root course module; the field uses the following format:  block-v1:{org}+{course_identifier}+{term}+ type@{mod_type}+block@{module_identifier}.  Example:  block-v1:MITProfessionalX+SysEngxB1+3T2016+ type@chapter+block@29cf9e4cb86d4ed0802f432bd65440c9 | String |
| mod_hex_id | The field captures the unique module identifier for the course modules, the 32 character alpha numeric string in the *id* field, with the exception of the root course module, which uses the *mod_hex_id* of “course”.  Example:  “29cf9e4cb86d4ed0802f432bd65440c9” | String |
| courseID | This field captures the course identifier for each module; the field uses the format: {org}+{course_identifier}+{term}.  Example:  “MITProfessionalX+SysEngxB1+3T2016” | String |
| mod_type | This field captures the type of edX course modules (or category) found in the course structure; Examples:   - course - chapter - sequential - vertical - video - html - openassessment | String |
| name | The text name of an edX course module presented to users enrolled in a course.  Example:  “Course Collaboration Tools (5 min)” | String |
| markdown | The markdown formatted text of question asked in problem modules during a course. | String |
| order | The sequential order of the module in the course; the order is computed from the course tree hierarchical. Module order is assigned based on a pre-order (NLR) depth first tree traversal. | Integer |
| childOrder | The field represents the order of course child modules in relationship to the parent subtree. Example:  chapter modules are children of the root course module in edX course structures; a course with 10 chapter modules would be given *childOrder* values sequenced from 1 to 10. | Integer |
| treelevel | The field indicates the level of the depth of the course module in the sub-tree of the course hierarchy. Values range from 0-4, with 0 representing for the root course node; and 4 representing course content modules, like videos, html pages, open assessment, and problems. | Integer |
| chpModPar | Provides the unique “chapter” module identifier for related sub-tree course modules. These identifiers come from the 1^st^ level of the course module hierarchy. | String |
| seqModPar | Provides the unique “sequential” module identifier for related sub-tree course modules. These identifiers come from the 2^nd^ level of the course module hierarchy. | String |
| vrtModPar | Provides the unique “vertical” module identifier for related sub-tree course modules. These identifiers come from the 3^rd^ level of the course module hierarchy. | String |
| parent | Provides the direct parent module identifier for related course modules. | String |
| modparent_childlevel | Combines the *parent* and *childOrder* fields into a unique identifier for module name look-ups in the edX event log data processing workflow. | String |

Note: A copy of the course structure documented in Table 2 is provided as Data A in S3 Dataset in comma separated value format (*dataA.csv*). Data A was used to create Fig 2A and 2B in the paper

**edX-2-learnerUserList.R data processing outputs**

The second script in the pipeline creates a composite user profile table from edX course database files *{org}-{course}-{run}-auth_user-{site}-analytics.sql* and *{org}-{course}-{run}-auth_userprofile-{site}-analytics.sql* files. Both of these files use a comma separated format.

Table 3 describes the composite dataset created by the processing script; these data files are detailed in the EdX Research Guide, section 6.3.3 User Data (<https://edx.readthedocs.io/projects/devdata/en/latest/internal_data_formats/sql_schema.html#user-data>).

**Table 3. Field list for {org}-{course identifier}-{term}-auth_user-learners.csv.**

| **Field Name** | **Description** | **Data Type** |
| --- | --- | --- |
| id | A unique identifier for a learner user in an edX course management system.  From *auth_user* data file. | Integer |
| is_staff | Most users have a 0 for this field. Set to 1 if the user is a staff member of edX.  From *auth_user* data file. | Boolean |
| is_active | This value is 1 if the user has clicked on the activation link that was sent to them when they created their account, and 0 otherwise.  From *auth_user* data file. | Boolean |
| is_superuser | Field indicates administrative access to Django forums. Value 1 set for site admins; 0 for all else.  From *auth_user* data file. | Boolean |
| last_login | A datetime of the user’s last login.  Format: YYYY-MM-DD hh:mm:ss  From *auth_user* data file. | Datetime |
| date_joined | A datetime of the user’s account was created.  Format: YYYY-MM-DD hh:mm:ss  From *auth_user* data file. | Datetime |
| gender | Categorical field indicating user gender.  Examples: m, f, o, (blank), NULL.  From *auth_userprofile* data file. | String |
| year_of_birth | User’s year of birth, collected at registration.  From *auth_userprofile* data file. | Integer |
| level_of_education | User’s level of education. Format is a categorical value.  From *auth_userprofile* data file. | String |
| goals | Collected during registration from a text field control with the label “Tell us why you’re interested in edX”  From *auth_userprofile* data file. | String |
| allow_certificate | Set to 1 (true) if a user has visited course site.  From *auth_userprofile* data file. | Boolean |
| country | A two digit country code based on the selection made by the learner during registration.  From *auth_userprofile* data file. | String |

**edX-3-eventLogExtractor.R data processing outputs**

The third script in the pipeline extracts a set of edX event tracking logs for each learner in the course, based on the log records’ associated user identifier (“id” field in Table 3). The logs are extracted from daily streaming JSON formatted logs.

Table 4 describes the event logs extracted by the script for further data processing. Extracted logs are saved as a comma separated value format. EdX event log data is described in the EdX Research Guide, section 6.7 Events in the Tracking Logs (https://edx.readthedocs.io/projects/devdata/en/latest/internal_data_formats/tracking_logs/index.html). This section of the EdX Research guide provides a:

- Example Log File in section 6.7.1.
- Common event log fields are described in section 6.7.2.
- Common types of learner events are described in section 6.7.3.

**Table 4. Field list for extracted learner edX event logs {user identifier}.csv.**

| **Field Name** | **Description** | **Data Type** |
| --- | --- | --- |
| agent | Provides agent information for event.  Example: *Mozilla/5.0 (Windows NT 6.1; WOW64; rv:52.0) Gecko/20100101 Firefox/52.0* | String |
| context.agent | Provides contextual information about learner’s action with content modules not captured in revised format. | JSON String |
| context.course_id | Course identifier in old log format. All fields use: *course-v1:MITxPRO+AMxB+1T2018* | String |
| context.course_user_tags | This field provides details on the browsers used by a user or the LMS server.  This field captures user agent data based on the old edX data format. The data field is minimally used in the AM course. When the field is used, it captures: an asides note and a module.usage_key that indicates the module id used with an event. | String |
| context.org_id | Organization identifier for EdX Platform host, all use: *MITxPRO* | String |
| context.path | Provides a contextual URL path to a piece of course content or events taken by learners. | String |
| context.user_id | EdX user identification number. | Integer |
| course_id | Course identifier used in revised log format, all use: *{org}+{course identifier}+{term}* | String |
| event | This field collects relevant data to describe a logged event. The data comes in JSON strings. | JSON String |
| event_source | Indicates if the event was generated by the learner user or the MITxPro system. The fields can be:   - server - browser - mobile | String Factor |
| event_type | This field is used to capture the type of event that a learner made during an interaction. These events can be of a variety of types. | String |
| host | Records the host of the course content. All records are *mitxpro.mit.edu*. | String |
| ip | This field captures the IP address of the user for each interaction | IP Address |
| session | Provides a session identifier for all browser events. Sever events lack the session identifiers. | String |
| name | Provides a free text name for event. The field lists names of all formal *event_types* for browser events, and provides NAs to events where *event_type* is listed as a URL string for server events. | String |
| page | This field is used to indicate a URL to a content module accessed by a user through a browser event, such as when they navigate to modules, accessing videos, and inputting responses to questions.  However, other records do not provide a URL and instead list “x_module”, which indicates that the server has logged a learner response in the system database, in particular this includes event_types.  Fields with “NA” values include server events for edx.grade.problem.submit events, and edx.forum.thread edx.forum.response and edx.forum.search events, and events involving server GET and POST actions (see fields *event_struct.GET* and *event_struct.POST*) | String |
| time | The date timestamp for an interaction event. Formatted:  YYYY-MM-ddTHH:mm:ss+TIMEOffest | Date Time |
| username | Full text username for a user/learner. This is removed for anonymity. | String |
| module_id | This field provides module identifiers for some browser events (with the exception of many video *event_types*) | String |
| event_struct. GET | This field indicates if a learner event was a GET action that requested data from the server. The field duplicates data found in the *event* field. The data is formatted as a JSON string. These events relate specifically to *event_source* “server” records, and *event_type* records. | JSON String |
| event_struct. POST | This field indicates if a learner event was a POST action that requested data from the server. The field duplicates data found in the *event* field. The data is formatted as a JSON string. These events relate specifically to *event_source* “server” records, and *event_type* records. | JSON String |
| event_struct. query | This field is used when a learner completes an action that is an *event_type* of “edx.forum.searched”. The field captures the text string used in the search. | String |
| event_struct. code | This field relates to video play, seek, and load events. These event types can have either categorical code: “hls” or a coded identifier, e.g. “  hQK5nGXc0UA” assigned | String |
| event_struct. new | This field represents is linked to navigation events (g,g seq_goto, seq_next, seq_prev) to indicate the sequential child module that a user is navigating to in an interaction. | Integer |
| event_struct. old | This field represents is linked to navigation events (g,g seq_goto, seq_next, seq_prev) to indicate the sequential child module that a user is navigating from in an interaction. | Integer |
| event_struct. current_time | This field is related to “speed_change_video” events for video modules. This field indicates the float time in secs when a speed change was made by a user. | Float |
| event_struct. currentTime | This field is related to “play_video” events for video modules. This field indicates the time in secs when a video was played by a learner. | Float |
| event_struct. answers | This field captures a learner’s response to the problem module question. Data is provided in a JSON string. | JSON String |
| event_struct. attempts | Provides the count of attempts that a learner has made for a given problem modules. | Integer |
| event_struct. success | This field is tied to the *event_type***: “**problem_check,” and is used to indicate if the learners’ response is *correct* or *incorrect.* | String  Factor |
| event_struct. grade | Provides the grade for a learner’s response to a problem module | Integer |
| event_struct. max_grade | Provides the maximum grade for a problem module. | Integer |
| event_struct. problem_id | This field provides a module identifier for the problem a learner interacts with in the course, which are indicated/tied to the *event_type***: “**problem_check,” and “save_problem_success.” | String |
| event_struct. state. correct_map | This field is used to indicate if a learner triggered a hint option for a problem module. This field is tied to the *event_type***: “**problem_check,” and “save_problem_success.” Data is provided in a JSON string.Only the “correctness” field of value. | JSON String |
| event_struct. state.input_state | This field is tied to the *event_type***: “**problem_check,” and “save_problem_success.” And provides a JSON string with a problem module’s answer identifier. | JSON String |
| event_struct. state.seed | Problem module answer state. All fields are given value of 1. | Binary (1/0) |
| event_struct. state. learner_answers | This field is tied to the *event_type***: “**problem_check,” and “save_problem_success.”  The field is used to indicate revised answer set after a learner receives a hint, indicated in the *event_struct***.***state.correct_map* field. This field is structured as a JSON string. | JSON String |
| event_struct. submission | This field is tied to the *event_type***: “**problem_check,” and is used to indicate if the learners’ response to specific course questions. This field is structured as a JSON string.  The initial string is an answer identifier to link common responses and to a given problem module identifier. Learner responses to course problems, collected in the file: *problem_analysis.json*; however, these responses to not indicate the text of the question or answer, as this field does. | JSON String |
| event_struct. type | This field is used to indicate the type of interaction of user events. Two example “types” were identified from learner logs:   - onSlideSeek - onCaptionSeek   These examples are tied to the event_type: “seek_video “ | String Factor |
| event_struct. new_time | This field is tied to the event_type: “seek_video,” and is used to indicate the time (secs) a user is targeting in a video. | Float |
| event_struct. old_time | This field is tied to the event_type: “seek_video,” and is used to indicate the time (secs) a learner stops watching a video to seek another portion of the video. | Float |

**edX-4-eventLogFormatter.R data processing outputs**

The fourth script in the pipeline processes each learner’s extracted edX event log dataset to a limited set of records that are the foundation of the learner trajectory network and analysis of learner activity in the course.

Table 5 describes the limited set of fields maintained from the original event log after processing by this script based on the list of user identifiers provided by a user.

**Table 5. Field list for processed learner edX event logs {user identifier}.csv.**

| **Field Name** | **Description** | **Data Type** |
| --- | --- | --- |
| user_id | User identifier for learner in edX course. | Integer |
| mod_hex_id | The unique module identifier for a course content module at the fourth level of the course structural hierarchy (a 32 character alphanumeric value). | String |
| order | The sequential order of the module in the course; the order is computed from the course tree hierarchical. Module order is assigned based on a pre-order (NLR) depth first tree traversal. | Integer |
| mod_parent_id | Provides the direct parent module identifier for related course modules. | String |
| module_type | This field captures the type of edX course modules (or category) found in the course structure.  Examples:   - video - html - problem - openassessment | String |
| event_type | This field captures the type of event that a learner or edX system generated in the event log. These responses vary depending on the system action or interaction with a given type of module.  See Common types of learner events are described in section 6.7.3 in the EdX Research Guide for more information  Event types preserved for this study include:  Some learner events do not have an event_type specified. In this case, the custom event “module_accessed” added to the record. | String |
| time | Datetime for when an action occurred in the event log. Formatted: Format: YYYY-MM-DD hh:mm:ss | Datetime |
| period | Calculated period between logged events. Events that are greater than or equal to an hour have temporal estimates revised to the median value of a given event_type of the event log record. | Float |
| session | EdX system session identifier associated with a learner and the logged event. These are not are updated when a learner logs in and out of the system, not when a learner times out after a period of inactivity. | String |
| tsess | Calculated sessions based on temporal period breaks that are greater than or equal to an hour. | Integer |
| event.attempts | Provides the count of attempts that a learner has made for a given problem modules. | Integer |
| event.grade | Provides the grade for a learner’s response to a problem module | Integer |
| event.max_grade | Provides the maximum grade for a problem module. | Integer |
| event.success | This field is tied to the *event_type***: “**problem_check,” and is used to indicate if the learners’ response is *correct* or *incorrect.* | String  Factor |

**edX-5-learnerTrajectoryNet.R data processing outputs**

The fifth script in the pipeline creates a trajectory network for each learner in the course, based on the processed edX event log produced by *edX-4-eventLogFormatter.R* script. For each learner, the script creates 1) a node and an edge lists as CSV formatted data files, and 2) a JSON formatted file that combines the node and edges list; an example of this output is Data B in S3 Dataset.

Table 6 describes the data fields for learner’s trajectory network node list, which includes module metadata and aggregate use statistics based on a learner’s event logs.

Table 7 describes the data fields for learner’s trajectory network edge lists, and documents transitions between content modules.

**Table 6. Field list for learner node list {user identifier}-nodes.csv.**

| **Field Name** | **Description** | **Data Type** |
| --- | --- | --- |
| mod_hex_id | The unique module identifier for a course content module at the fourth level of the course structural hierarchy (a 32 character alphanumeric value). | String |
| courseID | Course identifier used in revised log format, all use: *{org}+{course identifier}+{term}* | String |
| module.type | This field captures the type of edX course modules (or category) found in the course structure.  Examples include:   - video - html - problem - openassessment | String |
| desc | The name or textual description for a module. This is derived from the “name” field in a course structure, see Table 2. | String |
| L2 | Sequential module identifier parent of lower level course module. | String |
| L2label | The name or textual description for a module of the sequential module identifier parent of lower level course module. | String |
| L1 | Chapter module identifier parent of lower level course module. | String |
| L1label | The name or textual description for a module of the chapter module identifier parent of lower level course module. | String |
| order | The sequential order of the module in the course; the order is imputed from the course tree hierarchical. Module order is assigned based on a pre-order (NLR) depth first tree traversal. Field is taken from course structure, see Table 2. | Integer |
| unq_stu | The count of learners in the network tabulation. For individual learner trajectory networks, the value of this field is set to 1. | Integer |
| sessions | The count of unique values from the *session* field a given module in a learner’s processed event logs. | Integer |
| days | The count of unique days of a module used in a course, based on *time* field in a learner’s processed event logs. | Integer |
| events | The events value represents the count of interactions for a given module in a learner’s processed event logs. | Integer |
| totalTime | The total time a module is used during a course is based on the sum of *period* values in a learner’s processed event logs. | Float |
| progress_i | The number of inbound transitions, where a learner progresses from a low value *order* to one with a higher value *order*. Value is derived from the edge list of the learner trajectory network. | Integer |
| recurse_i | The number of inbound transitions, where a learner returns from a high value *order* to one with a low value *order*. Value is derived from the edge list of the learner trajectory network. | Integer |
| forward_o | The number of outbound transitions, where a learner moves forwards, i.e. from a module a with low value *order* to one with a higher value *order*. Value is derived from the edge list of the learner trajectory network. | Integer |
| backward_o | The number of outbound transitions, where a learner moves backwards, i.e. from a module a with higher value *order* to one with a lower value *order*. Value is derived from the edge list of the learner trajectory network. | Integer |
| sl | The count of self-loop events identified in the edge list of the learner trajectory network. This value indicates the number of times that a learner interacted with a module consecutively during the course. Value is derived from the edge list of the learner trajectory network. | Integer |
| attempts | The maximum value of *attempts* field in the processed learner event logs, indicates the number of attempts a learner interacted with a problem module. | Integer |
| max.grade | The maximum grade earned by a learner for a problem module is derived from the maximum value of the *event.grade* field in a learner’s processed event logs. | Integer |
| loads | The count of load events in the *event_type* field for a video module in a learner’s processed event logs. | Integer |
| load_time | Time spent on load events in the *event_type* field and sum of *period* field for a video module in a learner’s processed event logs. | Float |
| plays | The count of play events in the *event_type* field for a video module in a learner’s processed event logs. | Integer |
| play_time | Time spent on play events in the *event_type* field and sum of *period* field for a video module in a learner’s processed event logs. | Float |
| pauses | The count of pause events in the *event_type* field for a video module in a learner’s processed event logs. | Integer |
| pause_time | Time spent on pause events in the *event_type* field and sum of *period* field for a video module in a learner’s processed event logs. | Float |
| seeks | The count of seek events in the *event_type* field for a video module in a learner’s processed event logs. | Integer |
| seek_time | Time spent on seek events in the *event_type* field and sum of *period* field for a video module in a learner’s processed event logs. | Float |

**Table 7. Field list for learner edge list {user identifier}-edges.csv.**

| **Field Name** | **Description** | **Data Type** |
| --- | --- | --- |
| from | Module identifier for the source node in the learner trajectory network. Matches a value from the *mod_hex_id* field in the node list. | String |
| to | Module identifier for the target node in the learner trajectory network. Matches a value from the *mod_hex_id* field in the node list. | String |
| s.seqpos | Module order value for the source node in the learner trajectory network. This value should match the order value for the corresponding source module identifier found in the node list. Derived from the *order* field in the processed event logs. | Integer |
| s.session | The session identifier for an event that was logged to the source node in an edge list. This value matches a value in *session* field of a user’s processed event log. | String |
| s.tsession | The temporal session identifier for an event that was logged to the source node in an edge list. This value matches a value in t*sess* field of a user’s processed event log. | Integer |
| t.seqpos | Module order value for the target node in the learner trajectory network. This value should match the order value for the corresponding target module identifier found in the node list. Derived from the *order* field in the processed event logs. | Integer |
| t.session | The session identifier for an event that was logged to the target node in an edge list. This value matches a value in *session* field of a user’s processed event log. | String |
| t.tsession | The temporal session identifier for an event that was logged to the target node in an edge list. This value matches a value in t*sess* field of a user’s processed event log. | Integer |
| time | A datetime value for the source node event. Format is: YYYY-MM-DD hh:mm:ss | Datetime |
| dir | Indicates the direction of the transition in the sequential order between a source and target node (*t.seqpos* – *s.seqpos*). Positive values indicate forward transition are assigned “p”; negative values indicate backwards transitions are assigned “n”. | String |
| dis | Indicates the direction of the transition in the sequential order between a source and target node (*t.seqpos* – *s.seqpos*). Values range from positive and negative values. | Integer |
| sl | Indicates if a transition in the edge list is a self-loop, where the source and target nodes reference the same course modules. | Boolean |
| user_id | Identifies the learner event logs that were used to generate the edge list dataset. | String |

Note: The example of the learner trajectory networks found in Data B represents the kind of data used to create Fig 2C and 3 in the publication; these networks were visualized with Gephi v0.8.2.

**edX-6-moduleUseAnalysis.R data processing output**

The sixth script in the pipeline performs a module centered analysis of learners’ use of content and performance on assessments and activities in an edX course by combining learner node list statistics; this is typically run using a list of all active users in an edX course, see Data C in S3 Dataset (*dataC.csv*).

Script 6 may be used to process multiple cohorts from a course; Data E represents the combined data processing results for three cohorts from the analyzed edX course: all learners, learners earning a certificate, and learners that did not earn a certificate. Data C and Data E use common data fields that are described in Table 6.

Table 8 describes only those fields that are unique to Data C. Table 9 describes data fields that are unique to Data E.

The addition fields in represented in Data C, include:

1. the number of learners accessing the module during the course (i.e. *unq_stu*);
2. the proportion of learners that used a module (i.e. *prp_stu*);
3. the number of unique days the module was use (i.e. *days_ct*);
4. the number of unique sessions associated with a module (i.e. *session_ct*);
5. the overall average times and event counts (i.e. *avg_evt_stu*, *avgTimeStu*, *avgTimeEvt*); and
6. the average time and event counts for specific types of edX events found in the log (i.e. *avgAttempts, avgPointStu, maxPointsPrb, avgLoadEvents, avgLoadTime, avgPlayEvents, avgPlayTime, avgPauseEvents, avgPauseTime, avgSeekEvents,* & *avgSeekTime*).

**Table 8. Aggregate Learner Module Use Analysis (dataC.csv).**

| **Field Name** | **Description** | **Data Type** |
| --- | --- | --- |
| mod_hex_id | See field description in Table 6. | String |
| courseID | See field description in Table 6. | String |
| module.type | See field description in Table 6. | String |
| desc | See field description in Table 6. | String |
| L2 | See field description in Table 6. | String |
| L2label | See field description in Table 6. | String |
| L1 | See field description in Table 6. | String |
| L1label | See field description in Table 6. | String |
| order | See field description in Table 6. | Integer |
| unq_stu | The total count of learners that interacted with a module in the course, derived from a sum of all learner trajectory node lists. | Integer |
| prpstu | The proportion of learners that interacted with a module in the course. Field is calculated by dividing the *unq_stu* value by the total number of learner trajectory network node lists processed, in this case all active learners in the course. | Float |
| session_ct | The total count of unique values from the *session* field a given module, derived from a sum of all learner trajectory node lists. | Integer |
| days_ct | The total number of unique days of a module used in a course, derived from a sum of all learner trajectory node lists. | Integer |
| events | Total number of interaction events with a module based on the learner trajectory networks aggregated. See field description in Table 6. | Integer |
| avg_evt_stu | The average number of events per learner that used the module during the course; *events/unq_stu*. | Float |
| totalTime | Total time spent interacting with a module based on the learner trajectory networks aggregated. See field description in Table 6. | Float |
| avgTimeStu | The average time spent on a module by learners that used it during the course; *totalTime/unq_stu*. | Float |
| avgTimeEvt | The average time per event associated with a module; *totalTime/events*. | Float |
| progress_i | See field description in Table 6. | Integer |
| recurse_i | See field description in Table 6. | Integer |
| forward_o | See field description in Table 6. | Integer |
| backward_o | See field description in Table 6. | Integer |
| attempts | Sum total of learner attempts per problem module. See field description in Table 6. | Integer |
| avgAttempts | The average number of attempts per learner that submitted a response to a problem module during the course; *attempts/unq_stu*. | Float |
| points | The sum of the *max.grade* of learners earned for problem module. | Integer |
| avgPointStu | The average points earned by learners that interacted with a problem module; *points/unq_stu.* | Float |
| maxPointsPrb | The maximum value for the *max.*grade field of learner trajectory networks aggregated. See *max.grade* field in Table 6. | Float |
| loads | Total number of load interaction with a video module based on the learner trajectory networks aggregated. See field description in Table 6. | Integer |
| load_time | Total time spent load interactions with a video module based on the learner trajectory networks aggregated. See field description in Table 6. | Float |
| avgLoadEvents | The average number of load events with video modules for learners interacting with a module; *loads/­unq_stu.* | Float |
| avgLoadTime | The average time spent on a load events for video module by leaners that used it during the course; *load_time/unq_stu*. | Float |
| plays | Total number of play interaction with a video module based on the learner trajectory networks aggregated. See field description in Table 6. | Integer |
| play_time | Total time spent play interactions with a video module based on the learner trajectory networks aggregated. See field description in Table 6. | Float |
| avgPlayEvents | The average number of play events with video modules for learners interacting with a module; *plays/­unq_stu.* | Float |
| avgPlayTime | The average time spent on a play events for video module by leaners that used it during the course; *play_time/unq_stu*. | Float |
| pauses | Total number of pause interaction with a video module based on the learner trajectory networks aggregated. See field description in Table 6. | Integer |
| pause_time | Total time spent pause interactions with a video module based on the learner trajectory networks aggregated. See field description in Table 6. | Float |
| avgPauseEvents | The average number of pause events with video modules for learners interacting with a module; *pauses/­unq_stu.* | Float |
| avgPauseTime | The average time spent on a pause events for video module by leaners that used it during the course; *pause_time/unq_stu*. | Float |
| seeks | Total number of seek interaction with a video module based on the learner trajectory networks aggregated. See field description in Table 6. | Integer |
| seek_time | Total time spent seek interactions with a video module based on the learner trajectory networks aggregated. See field description in Table 6. | Float |
| avgSeekEvents | The average number of seek events with video modules for learners interacting with a module; *seeks/­unq_stu.* | Float |
| avgSeekTime | The average time spent on a seek events for video module by leaners that used it during the course; *seek_time/unq_stu*. | Float |

**Table 9. Unique Data Fields for Comparison of Cohort Module Use Statistics (dataE.csv).**

| **Field Name** | **Description** | **Data Type** |
| --- | --- | --- |
| Note | Fields 1-9 are described above in Table 6. |  |
| unq_stu{.1 to .n} | The total count of learners that interacted with a module in the course, derived from a sum of all learner trajectory node lists.  Columns are duplicated for the number of cohorts that are being compared. | Integer |
| events{.1 to .n} | Total number of interaction events with a module based on the learner trajectory networks aggregated. See field description in Table 6.  Columns are duplicated for the number of cohorts that are being compared. | Integer |
| totalTime{.1 to .n} | Total time spent interacting with a module based on the learner trajectory networks aggregated. See field description in Table 6.  Columns are duplicated for the number of cohorts that are being compared. | Float |

Note: Sample Data E was used to create cohort groups and labels for Figs 4 and 5.

**edX-7-studentFeatureExtraction.R data processing output**

The seventh script in the pipeline produces an analysis interaction and performance analysis for each active learners’ for the length of the overall course, which are combined into a single data set for visualization. Data D in S3 Dataset (*dataD.csv*) represents the results of this script for the course examined in the paper.

Table 10 describes the fields in Data D output by the processing script. The fields *user_id*, *gender, yob, loe* are excluded from the supplemental data file to anonymize the learner data that is shared with the project.

**Table 10. Field List for Aggregate Analysis of Learner Activity (dataD.csv).**

| **Field Name** | **Description** | **Data Type** |
| --- | --- | --- |
| user_id | A unique identifier for a learner user in an edX course management system.  From *auth_user* data file. | Integer |
| grade | The final grade in the course, taken from *grade* field in table {org}-{course identifier}-{term}-certificates_generatedcertificate-prod-analytics.sql found in the course edX data package. | Float |
| cert_status | The final grade in the course, taken from *cert_status* field in table {org}-{course identifier}-{term}-certificates_ generatedcertificate-prod-analytics.sql found in the course edX data package. | String |
| gender | Categorical field indicating user gender.  Examples: m, f, o, (blank), NULL.  From *auth_userprofile* data file. Field excluded from sample data. | String |
| yob | User’s year of birth, collected at registration.  From *auth_userprofile* data file. Field excluded from sample data. | Integer |
| loe | User’s level of education. Format is a categorical value. From *auth_userprofile* data file. Field excluded from sample data. | String |
| sessions | The count of unique values from the *session*s in a learner’s processed event logs. | Integer |
| days_unq | The count of unique days of a learner was active in a course, based on *time* field in a learner’s processed event logs. | Integer |
| mods_unq | The count of unique modules that a learner used during a course, derived from a learner’s processed event logs. | Float |
| vid_mods | The count of unique video modules that a learner used during a course, derived from a learner’s processed event logs. | Integer |
| prb_mod | The count of unique problem modules that a learner used during a course, derived from a learner’s processed event logs. | Integer |
| oa_mods | The count of unique open assessment modules that a learner used during a course, derived from a learner’s processed event logs. | Integer |
| events | The count of events that a learner made during a course, derived from a learner’s processed event logs. | Integer |
| vid_events | The count of events that a learner made with video modules during a course, derived from a learner’s processed event logs. | Integer |
| prb_events | The count of events that a learner made with problem modules during a course, derived from a learner’s processed event logs. | Integer |
| oa_events | The count of events that a learner made with open assessment modules during a course, derived from a learner’s processed event logs. | Integer |
| oa_peerAccessEvents | The count of peer assessment events that a learner made with open assessment modules during a course, derived from a learner’s processed event logs. | Integer |
| oa_getPeerEvents | The count of getting peer assessment events that a learner made with open assessment modules during a course, derived from a learner’s processed event logs. | Integer |
| seqNextEvents | The count of “seqNext” transition events that a learner made with during a course, derived from a learner’s processed event logs. | Integer |
| seqPrevEvents | The count of “seqPrev” transition events that a learner made with during a course, derived from a learner’s processed event logs. | Integer |
| seqGotoEvents | The count of “seqGoto” transition events that a learner made with during a course, derived from a learner’s processed event logs. | Integer |
| modAccessEvents | The count of “modAccess” events that a learner made with during a course, derived from a learner’s processed event logs. | Integer |
| total_time | The total time spent active in the course based on the some of the *period* field from a learner’s processed event logs. | Float |
| vid_time | The total time spent active on video modules in the course based on the some of the *period* field from a learner’s processed event logs. | Float |
| prb_time | The total time spent active on problem modules in the course based on the some of the *period* field from a learner’s processed event logs. | Float |
| oa_time | The total time spent active on open assessment modules in the course based on the some of the *period* field from a learner’s processed event logs. | Float |
| oa_peerAccessTime | The total time spent active on peer assessments events in the course based on the some of the *period* field from a learner’s processed event logs. | Float |
| oa_getPeerTime | The total time spent active on getting their peer assessments in the course based on the some of the *period* field from a learner’s processed event logs. | Float |
| seqNextTime | The total time spent active on “seqNext” transition event type in the course based on the some of the *period* field from a learner’s processed event logs. | Float |
| seqPrevTime | The total time spent active on “seqPrev” transition event type in the course based on the some of the *period* field from a learner’s processed event logs. | Float |
| seqGotoTime | The total time spent active on “seqGoto” transition event type in the course based on the some of the *period* field from a learner’s processed event logs. | Float |
| modAccessTime | The total time spent active on “modAccess” transition event type in the course based on the some of the *period* field from a learner’s processed event logs. | Float |
| prb_attempts | The count of all problem submission events field from a learner’s processed event logs. | Integer |
| prb_correct | The total points earned in problem submission events by of the sum of *event.grade* field from a learner’s processed event logs. | Integer |
| prb_totalPoints | The total points possible from submission events, based on of the sum of *event.max_grade* field from a learner’s processed event logs. | Integer |

Note: Data D was used to create cohort groups and labels in Figs 4 and 5, and was used to create all elements of Fig 6 in the paper.
